# KLHL5 knockdown increases cellular sensitivity to anticancer drugs

**DOI:** 10.18632/oncotarget.26462

**Published:** 2018-12-21

**Authors:** Robert J. Schleifer, Shuchun Li, Wyatt Nechtman, Eric Miller, Shan Bai, Ashok Sharma, Jin-Xiong She

**Affiliations:** ^1^ Center for Biotechnology and Genomic Medicine, Medical College of Georgia, Augusta University, Augusta, GA 30912, USA; ^2^ Department of Biostatistics and Epidemiology, Medical College of Georgia, Augusta University, Augusta, GA 30912, USA; ^3^ Department of Obstetrics and Gynecology, Medical College of Georgia, Augusta University, Augusta, GA 30912, USA

**Keywords:** KLHL, KLHL5, cell cycle, combined therapy, synergistic effects

## Abstract

KLHL family genes are noted for their involvement in the E3 ligase ubiquitination pathway through binding with Cullin-3 (CUL3) resulting in degradation of specific binding partners. KLHLs are thus intriguing genes for cancer as they can directly influence the degradation of therapeutically relevant cell cycle regulators such as Aurora Kinase, PLK1, or CDK1. However, most KLHL family members remain understudied within the literature. This study explores the relationship of expression of KLHL member, *KLHL5*, with the pharmacologic effect of anti-cancer drugs. *KLHL5* knockdown decreased the proliferation and viability of cancer cells and sensitized cancer cells to numerous anti-cancer drugs. Drugs related to cell cycle including Akt/PI3K/mTOR inhibitors were especially sensitized by *KLHL5* knockdown. The potential of KLHL5 as a prognostic or diagnostic cancer marker was compared to other KLHLs through a pan-cancer study of The Cancer Genome Atlas (TCGA) tumor groups. While KLHL5 expression shows marginal dysregulation in cancer, other KLHLs exhibit significant dysregulation in all cancer types, and exceptionally in renal carcinomas. This study advocates for further study of KLHLs as potential alternative therapeutic targets, since while *KLHL5* is a novel gene impacting anticancer drug effects, others may have a similar impact on drug effect while having greater potential as diagnostic or prognostic markers.

## INTRODUCTION

The KLHL family genes interact in signal transduction mechanisms including protein degradation through the ubiquitin ligase system, actin dynamics, and cell-cycle regulation [1]. KLHLs bind to and mark specific substrates for degradation as a consequence of sequence variance in the BTB (Broad Complex, Tramtrack, and Bric à Brac) domain [1]. This occurs through the E3 ubiquitin ligase complex where KLHLs bind to Cullin-3 (CUL3) [2]. Possession of BACK domains and namesake Kelch motifs distinguish KLHLs from other BTB proteins [3]. The Kelch motif associates with actin allowing the organization of organelles, cytoskeleton, and the plasma membrane [4].

KLHLs serve as regulators of critical cellular pathways including cell cycle. KLHL22 regulates the mitotic kinase PLK1 (Polo-like kinase 1) and helps control of the G2/M checkpoint [5]. KEAP1 (KLHL19) regulates degradation of the cytoprotective Nrf2, a transcription factor associated with oxidative stress pathways [6]. KLHL21 regulates the mitotic kinase involved in the spindle assembly checkpoint Aurora B [7], promotes cell migration and focal adhesion dynamics [8], and regulates NF-κB signaling through inhibition of IKKβ [9]. Higher expression of KLHL21 is associated with poor hepatocellular carcinoma (HCC) prognosis [10]. KLHL12 regulates the Wnt-beta-catenin pathway through degradation of Dishevelled [11]. KLHL20 complexes with DAPK (Death Associated Protein Kinase) and can be a determining factor of interferon (IFN)-induced cell death [12]. While these examples show a diversity of functions and potential importance in cancer disease progression or therapeutic response, the functional roles of many KLHLs remain unstudied.

We selected *KLHL5* to explore its potential as a primary or combinatorial therapeutic target or biomarker. The full function of *KLHL5* has not been elucidated, but annotations associate it with cell cycle regulars including Aurora B and PLK1. Upon initial cloning, KLHL5 showed relatively higher expression in ovarian, adrenal, and thyroid tissues [13]. It also possessed a splicing variant naturally expressed in human fetal brain tissue [14]. Targeting KLHL5 may offer an alternative method to modulate cell cycle activity, a popular anti-cancer strategy showing clinical potential in single treatment with inhibitors such as Barasertib [15–17] or with the ability to overcome drug resistance or treat refractory disease when used in combinatorial therapies [18–20]. Deshmukh *et al*. have recently outlined the potential of targeting KLHL family member KEAP1 as a therapeutic target in cancer and neurodegenerative diseases because of its association with the NRF2 pathway [21]. In addition, a KEAP1 mutation was shown by Shibata *et al*. to directly influence chemoresistance in gallbladder cancer [22]. We believe that other KLHL members may also impact drug sensitivity, response to therapy, chemoresistance, or have potential as therapeutic targets.

The goals of this study are: 1) to explore the relationship of *KLHL5* expression to drug effect. 2) Assess the potential of *KLHL5* as a target for combinatorial anticancer treatment. 3) To investigate the dysregulation and prognostic implications of KLHL5 as compared to other KLHLs in human cancer.

## RESULTS

### KLHL5 expression correlates with decreased drug sensitivity of numerous compounds

Pearson correlation values associating drug sensitivity and gene expression across NCI-60 cell lines were utilized to identify *KLHL5* as a candidate for impacting drug response. Among the ~20,000 anti-cancer compounds in the NCI dataset, over 1600 compounds had a negative correlation less than -0.400 between drug effect and *KLHL5* expression (Supplementary Figure 1). The compounds within this collection include a wide variety of compounds with known anticancer activity from clinical drugs to synthesized or natural compounds with limited study. The high number of negatively correlated compounds indicates that several compounds tended to be less potent against cell lines with higher *KLHL5* expression. Other KLHLs did not have as many compounds with negative correlations making *KLHL5* of particular interest (Supplementary Figure 1).

### KLHL5 knockdown reduced cancer cell proliferation

**Figure 1:**
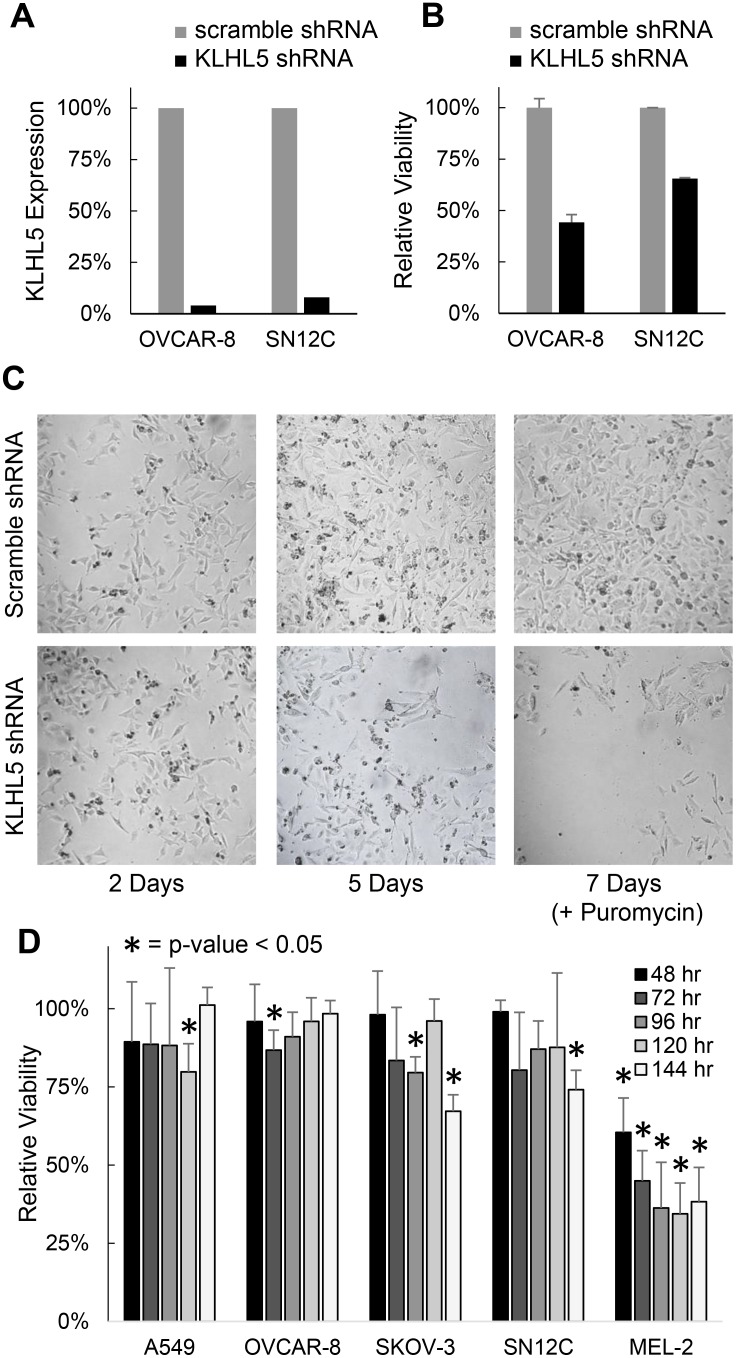
KLHL5 knockdown decreases cancer proliferation or viability (**A**) Confirmation of KLHL5 knockdown efficacy using qRT-PCR. (**B**) Relative viability of OVCAR-8 and SN12C cells 72 hours after shRNA knockdown of KLHL5. (**C**) Representative imagery of KLHL5 knockdown in OVCAR-8 cells over time. Puromycin was added at day 5 to ensure selection for cells with successful transfection (**D**) Viability of cells over time after KLHL5 knockdown compared to scramble control. Data represents the mean of three replicates. Asterisks mark *p*-values from a two-tailed *t*-test that are < 0.05.

Two knockdown technologies were utilized to observe the impact of *KLHL5* expression. Of the five cell lines tested OVCAR-8 (ovarian adenocarcinoma) and SN12C (renal cell carcinoma) were the most tolerant and or capable of knockdown with both the shRNA assay and the siRNA assay. With shRNA knockdown, these cell lines showed strong knockdown efficacy, as confirmed by qRT-PCR with the expression of *KLHL5* reduced by 96% in OVCAR-8 and 92% in SN12C at a 72 hour time point (Figure 1A). Knockdown corresponded with a decrease in both SN12C and OVCAR-8 growth rates (Figure 1B), with continued suppression of cell growth among the knockdown cells (Figure 1C).

To impact of *KLHL5* knockdown was also studied using a lipofectamine delivered siRNA-based knockdown system. A major difference between the siRNA and shRNA systems is that shRNA once integrated into the genomic DNA will constitutively express inhibitory RNA, while the siRNA approach is more transient. The siRNA knockdown had a less pronounced impact on cell proliferation than shRNA, but the decreases in viability were still statistically significant (*p* < 0.05) (Figure 1D). *KLHL5* knockdown decreased viability versus scramble control in all five cell lines with the most profound decrease in the melanoma cell line MEL-2. Growth rate appeared to stabilize or recover after 72 hours in cell lines such as OVCAR-8. Ultimately, knockdown using either knockdown technology resulted in a decrease in cellular proliferation or after *KLHL5* knockdown. The siRNA technology was more suitable for concurrent treatment with drugs since the conditions were less harsh upon normal cellular growth.

### KLHL5 knockdown synergizes with cell cycle inhibitors and Akt/PI3K/mTOR inhibitors

**Figure 2:**
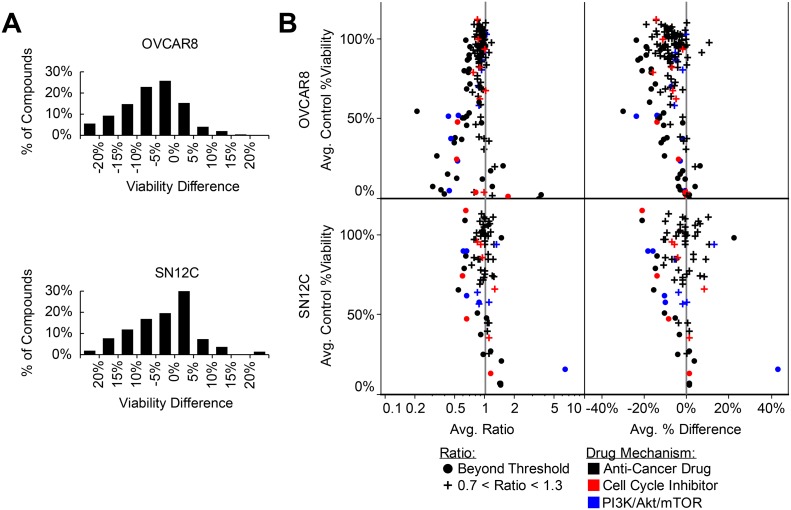
KLHL5 knockdown sensitizes to anticancer drugs (**A**) Viability change for paired library screening of anticancer compounds. The difference reflects the normalized observed viability minus the expected viability. Data represents the average distance between the means of two replicates. (**B**) Drug sensitivity shifts with KLHL5 knockdown by ratio and difference. Observations represented with a round symbol are distinguished for those ratios of greater 1.3 or less than 0.7 (common thresholds for synergistic or antagonistic relationships). Plus symbols are used for relationships where little change in sensitivity was observed. Color is used for drugs targetting cell cycle (red) or PI3K/Akt/mTOR pathways (blue). Data represents the mean of two replicates.

Cellular viability was observed with treatment of 346 anti-cancer compounds in the presence and absence of *KLHL5* siRNA knockdown. This was compared to viability expected for combinatorial treatment with each drug and KLHL5 (Expected viability calculated as the product of the observed viability for knockdown only and drug only: EV = V_kd_ x V_Drug_). Exposure to *KLHL5* knockdown increased the sensitivity to numerous anticancer drugs in tests on both SN12C and OVCAR-8 cell lines (Figure 2A). Synergistic properties were calculated for each drug by differences viability as well as in the ratio of expected to observed cells (Figure 2B). Cell cycle inhibitors and inhibitors of the PI3K/Akt/mTOR pathway were frequently potentiated by *KLHL5* knockdown (Figure 2B). The PI3K inhibitors with strong combinatorial synergy included GDC-0941, XL147, YM201636, and ZSTK474. The mTOR inhibitors that synergized included AZD8055, CH5132799, Everolimus (Rapamycin), INK 128, and Ku-0063794. The Akt inhibitor, Triciribine, had strong but inconsistent results between cell lines with synergy with *KLHL5* knockdown in OVCAR-8 cells but antagonism in SN12C cells (Supplementary Figure 2). Cell cycle inhibitors that inhibited checkpoint, Aurora Kinase, or Cdks were also among compounds that observed synergistic anti-cancer effect with *KLHL5* knockdown. Aurora Kinase inhibitors with synergism included AMG 900, Barasertib, CYC116, Danusertib, ENMD-2076, PF-03814735, SNS-314 Mesylate, and Tozasertib (Supplementary Figure 2).

### KLHL5 knockdown decreases the required dosage of anticancer compounds

**Figure 3:**
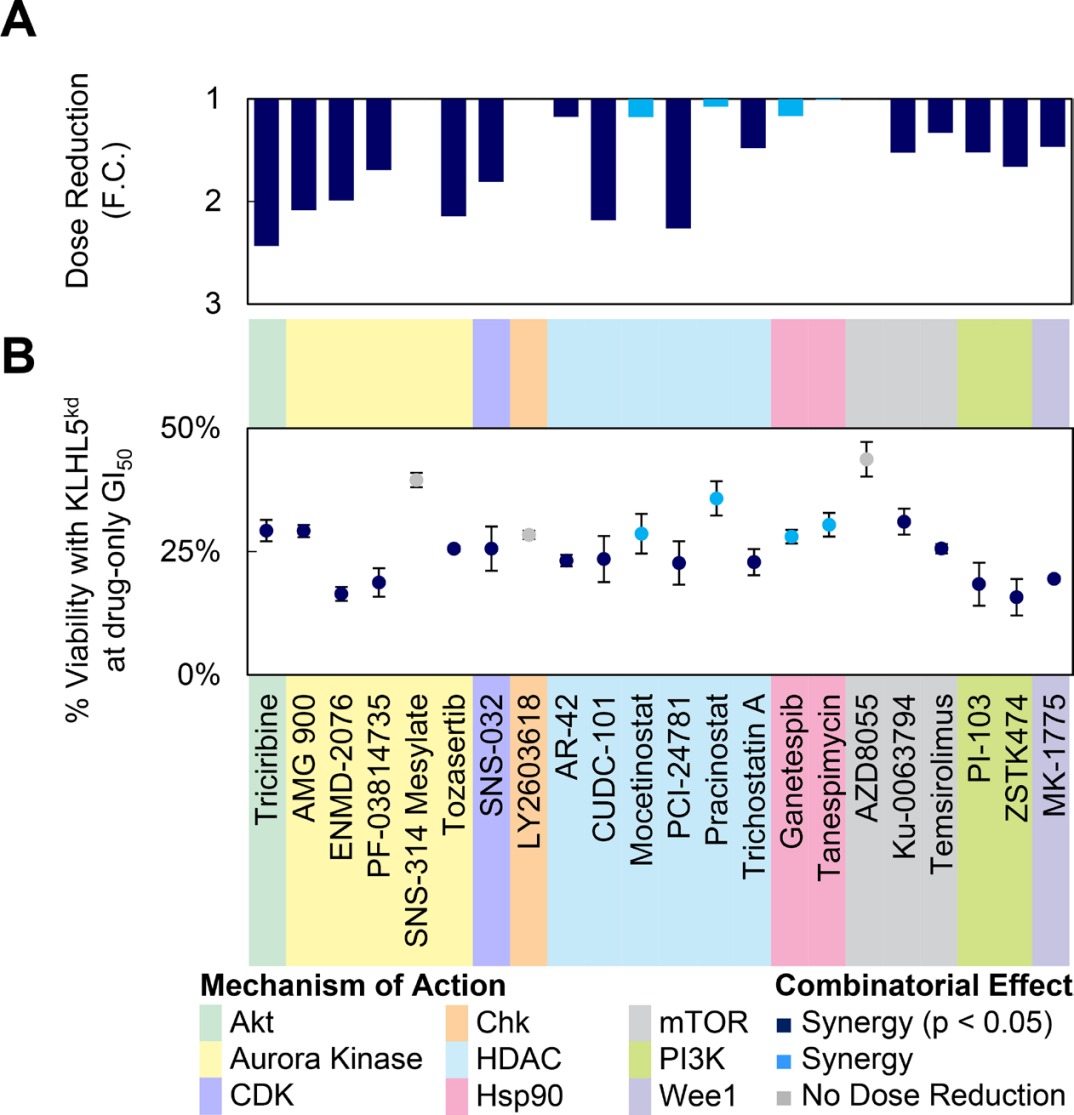
Dose reduction by KLHL5 knockdown (**A**–**B**) GI50 values were calculated from serial dilution curves for several anticancer compounds. In this experiment, KLHL5 knockdown alone resulted in 78.7% ± 2.2% viability. Viabilities were normalized to 100% to account for the effect of knockdown-only on viability. Data represent means ± s.d. from three replicates. (A) The dose reduction of the GI50 is represented as a fold change. (B) The viability for the combination of knockdown and drug at the GI50 concentration observed with drug treatment only.

Drugs with combinatorial synergy to *KLHL5* knockdown were investigated further by titration screening (Figure 3). Two metrics measuring combinatorial effect were calculated to characterize the titration curves. First, the drug concentration required to achieve the same viability (50% growth inhibition) (Figure 3A). Second, cellular viability at the concentration required to achieve 50% growth inhibition (GI_50_) for drug-only treatment (Figure 3B). The majority of the cell cycle inhibitors or PI3K/Akt/mTOR pathway inhibitors a two-fold dose reduction at drug-only GI_50_ (Figure 3A). It should be noted that several of the compounds that showed significant differences in viability did not also have strong dose reduction (FC) (Figure 3B). This discrepancy can be due to a shift in the steepness of the slope of the titration curve.

### KLHLs are dysregulated in cancer vs. normal tissue

**Figure 4:**
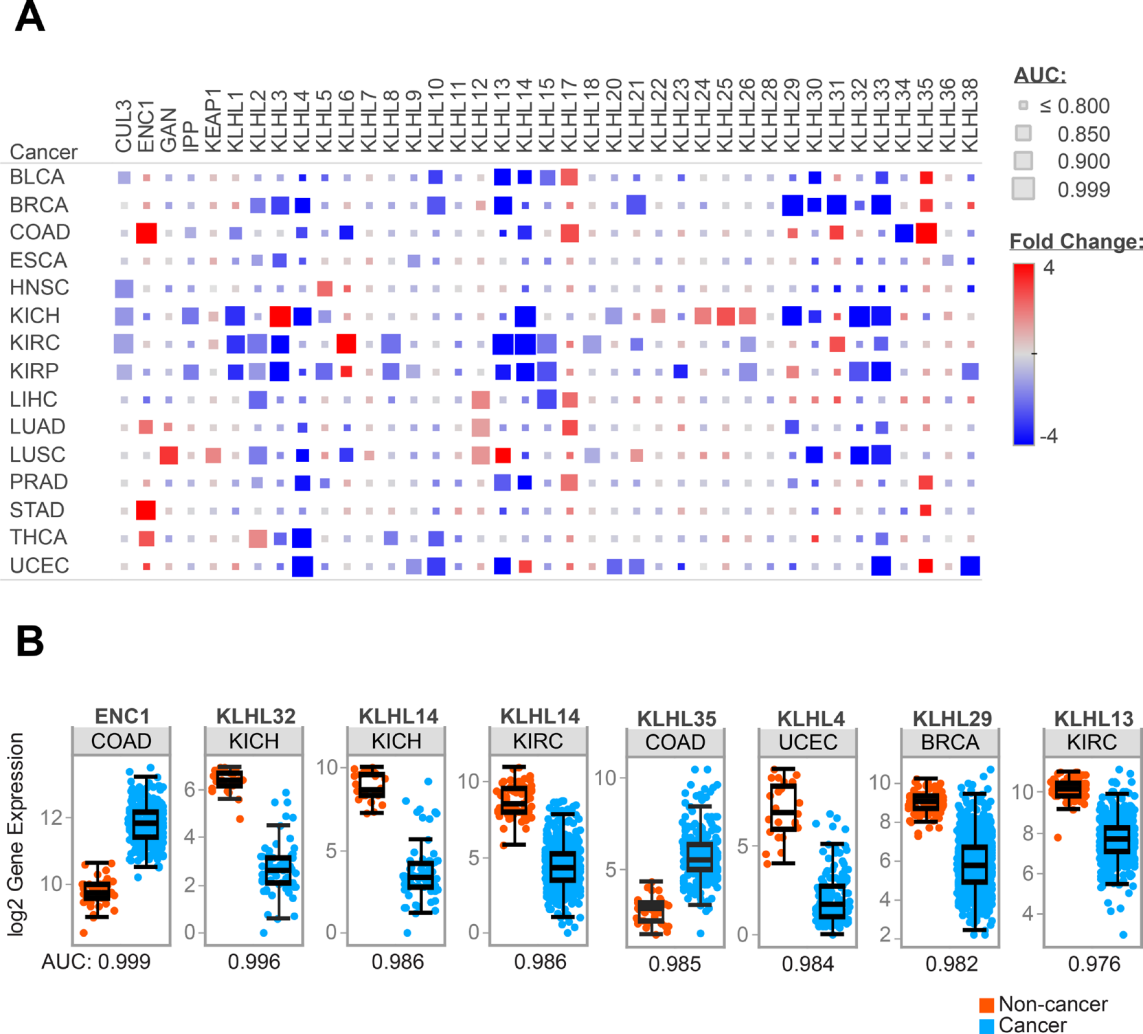
KLHL expression differences in cancer Representation of RNAseq data for fifteen TCGA tumor types (**A**) Heatmap of expression differences between cancer and adjacent normal tissue for KLHLs in the TCGA data. Size of each block reflects AUC from ROC curves comparing cancer vs. normal. (**B**) Comparison of gene expression in cancer vs. normal for specific KLHLs in TCGA tumor types which demonstrated high AUC values from ROC analysis.

The potential clinical implications of *KLHL5* were explored using a pan-cancer study of KLHL expression in fifteen cancers from The Cancer Genome Atlas (TCGA). Expression differences between tumor and adjacent normal were studied in cancers where data from ten or more patients was available. These differences were characterized by fold change (FC), *p*-value, and area under the curve (AUC) for the receiver-operator characteristic (ROC). Twenty-five KLHLs had an AUC of greater than 0.9 on at least one of the fifteen TCGA cancer types (Figure 4A). Twelve KLHLs showed greater than 4-fold expression differences in two or more cancers. Eight of these had decreased expression in cancer (KLHL3, KLHL4, KLHL13, KLHL14, KLHL29, KLHL30, KLHL32, and KLHL33) while four genes (ENC1, KLHL12, KLHL17, and KLHL35) had patterns of up-regulated gene expression. Across the panel, significant gene expression decreases (*p* < 0.05, FC>|2|) outnumbered increases by 106 to 36.

Expression differences in KLHLs were cancer-type specific. KLHL expression was most dysregulated in BRCA, COAD, KICH, KIRC, KIRP, and UCEC. Cancers such as ESCA, HNSC, PRAD, and STAD had few KLHLs with expression differences. Renal cancers featured the most dramatic expression differences, in all three types studied (KICH, KIRC, KIRP). The chromophobe subtype (KICH) appeared to have a distinct pattern of KLHL dysregulation compared to clear cell (KIRC) or papillary (KIRP) subtypes. Cullin-3 (CUL3) which complexes with many KLHLs while recruiting targets for E3 ubiquitin ligase did not comparatively have a high fold-change in gene expression. While not profound in fold-change, the expression of CUL3 was largely downregulated in cancer vs. normal with significant down-regulation in five TCGA groups: KIRC, KICH, KIRP, HNSC, and BLCA. The eight strongest predictive values (AUC > 0.975) for KLHLs with a TCGA cancer type as are shown in Figure 4B.

*KLHL5* expression dysregulation was not pronounced compared to other KLHLs. Interestingly *KLHL5* was one of only two KLHLs with expression changes in head and neck squamous cancer (AUC = 0.864). *KLHL5* also had a significant expression decrease in renal papillary (AUC = 0.899), but largely the diagnostic value of expression differences as indicated by AUC curves from these cancer types was underwhelming.

### Prognostic implications of KLHL expression are more prominent in renal cancers

**Figure 5:**
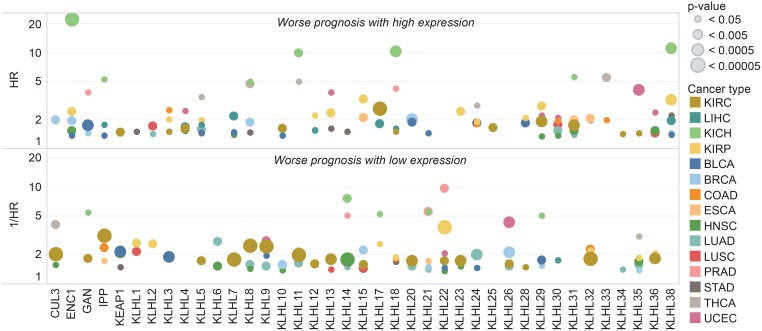
KLHL expression associated with prognostic differences Patients with high and low expression of each gene were compared by survival within each TCGA tumor type. Kaplan–Meier survival curves and hazard ratios were calculated for each gene in each cancer separating the patient cohort into two groups at ten percentile increments. Shown are the strongest hazard ratios for any gene-cancer type pair where a significant survival difference *p* < 0.05 was observed.

Patients within each cancer type were grouped by high and low expression of each KLHL to observe survival differences indicated by Cox proportional hazards models (Figure 5). Four trends became apparent upon survival analysis. 1) A survival difference associated with differential KLHL expression is present for most KLHLs in at least one cancer type. 2) Expression differences of most KLHLs associated with survival differences in KIRC (renal clear cell carcinoma). 3) All cancer types featured at least one significant prognostic association with a KLHL gene. 4) KICH (Chromophobe renal cell carcinoma) featured the most extreme hazard ratios for patient prognosis in association with KLHL expression.

*KLHL5* had two significant hazard ratios: (HR >2 or 1/HR >2 and *p*-value <0.05) with only THCA (HR = 3.47) and significant inverse hazard ratio with only PRAD (1/HR = 6.60). Eighty-one significant hazard ratios or inverse hazard ratios were observed across all KLHLs. Only *KLHL29*, *KLHL38*, and *KLHL22* had hazard ratios or inverse hazard ratios >2 in the same direction with three cancer types. *KLHL29* (HR>2) in KIRP, LIHC, and UCEC. *KLHL38* (HR >2) in KICH, KIRP, and STAD. *KLHL22* (1/HR>2) with KIRP, PRAD, and UCEC. KLHLs with significant hazard ratios or inverse hazard ratios for four cancer types regardless of direction were *KLHL14*, *KLHL22*, *KLHL29*, *KLHL32*, and *KLHL36*.

## DISCUSSION

This study provides evidence that KLHL5 expression decrease increase sensitivity to anticancer compounds, especially Akt/PI3K/mTOR inhibitors. This implies that inhibiting or regulating KLHL5 could be a potential method of sensitizing cancer cells to specific drugs. Because of this relationship to anti-cancer therapeutics, *KLHL5* upregulation could theoretically lead to resistance to therapeutics as a drug resistance gene. The TCGA clinical data offers limited evidence that *KLHL5* has consistent dysregulation or impact on patient prognosis. The interaction of KLHL5 with anti-cancer compounds that target cell cycle processes is consistent with the known and suspected functions of *KLHL5* as visualized from the STRING database (Supplementary Figure 3). The proteins associated with KLHL5 are enriched for in ontologies of cell cycle (*p* = 3.75E-30) and protein ubiquitination (*p* = 4.12E-42). From our study of *KLHL5* knockdown, the reduction of *KLHL5* expression led to a decrease in cancer cell proliferation and viability. While a therapeutic strategy targeting *KLHL5* may have some potential, it may have challenges as KLHL5 is constitutively expressed in several normal tissues. The potential of KLHL5 as a target is speculative, as a side-effect profile or physiological impact of a theoretical KLHL5 inhibitor or expression modulator is impossible to predict from current knowledge of the gene's function.

KLHLs may be uniquely positioned as potential anti-cancer targets by facilitating ubiquitination of genes associated with cell cycle, stress response, and protein trafficking pathways. While several KLHLs are known to influence tumor progression through the marking specific substrates for destruction, the substrates for the majority of KLHLs remain a frontier for discovery. This comprehensive investigation of KLHLs across fifteen cancer types encourages further investigation of these genes as prognostic or diagnostic biomarkers. The high AUC values observed in ENC1 in colon, KLHL32 in renal chromophobe, or KLHL14 in renal chromophobe or renal clear cell present compelling candidates for further exploration of the relationship of each KLHL on tumorigenesis and disease progression or their potential as a diagnostic biomarker.

Prognostic differences were especially noteworthy for KLHL expression in renal cancer. High hazard ratios were noted in chromophobe subtype (KICH) on expression differences of ENC1, KLHL8, KLHL18, KLHL31, or KLHL38 (Figure 5). Interestingly, renal clear cell carcinoma (KIRC) and renal papillary (KIRP) had a distinct from KICH had dysregulation of the E3 ligase system from the reduced expression of *CUL3*. KLHL-CUL3 dysregulation may be associated with a higher level of cell cycle deregulation among the patients within that group. Cell cycle deregulation is observed in all cancers at varying prevalence, and profoundly impacts prognosis and clinical response. Several other KLHLs may have clinical relevance based on the TCGA patient data. We believe that exploration of the relationship between gene expression and the impact of anti-cancer drugs will provide additional insight for their potential as therapeutic targets to control cell cycle dysregulation. Additionally, further knowledge of the substrates for KLHLs and relationship to the E3 ligase system would be valuable information for the scientific community. We believe that several KLHLs are likely to represent novel, non-conventional therapeutic targets with untapped potential as markers of prognostic or diagnostic differences in cancer.

## MATERIALS AND METHODS

### Cell culture

OVCAR-8, A549, SN12C, MEL-2, and SKOV-3 cell lines were obtained from the DTP, DCTC Tumor Repository, and confirmed with DNA fingerprinting. Cell lines were cultured in 1640-RPMI (Lonza) with 10% Fetal Bovine Serum (Sigma), and 1% Corning™ cellgro™ Antibiotic-Antimycotic Solution (Thermo Fisher) with 100 IU Penicillin, 100 μg/ml of streptomycin, and 250 ng/ml of Amphotericin B at 37° C with 5% CO_2_. Experiments using 96-well plates utilized inner wells only with outer wells filled with PBS to minimize the effects of evaporation. Experiments using OVCAR-8 cells were performed within five passages from passage six, which was the passage obtained from DTP. Authentication of cell identity was performed using STR profiling and matched to known profiles in online databases. Cell lines tested negative for mycoplasma using Universal Mycoplasma Detection Kit (ATCC^®^ 30–1012K™) (ATCC, Manassas, Virginia, USA) following vendor protocols.

Cell counts were estimated using Cell Counting Kit-8 (CCK-8) (Dojindo Molecular Technologies, Rockville, Maryland, USA). Optical density at 450 nm was determined using a multifunctional microplate reader (Bio-Tek). Cell counts were normalized by setting cell-free negative control to 0% and control growth to 100% (*n* = 3). Control growth was determined from cells treated with vehicle only on the same plate as experimental conditions to minimize plate-to-plate variation.

### Drug activity assays

A library of 346 FDA-approved drugs or clinical trial anticancer compounds was screened at a concentration of 100nM (Selleckchem, Houston, Texas, USA). Compounds were diluted and stored in DMSO prior to treatment of 96-well plates. Compounds were delivered in 1 μL drug into 99 μL of media to achieve the indicated final concentrations. Comparisons of drug effect were performed using data generated on the same day. A minimum of two replicates were performed with each gene knockdown-drug pair at each concentration in library screening, and a minimum of three replicates for all other tests. Significance was determined using a 0.05 *p*-value as calculated using a Student's *T*-test (two-tailed, unpaired). The GI_50_ (Growth Inhibition 50%) concentrations were calculated from viability curves using normalized viability data (SigmaPlot).

### Gene knockdown

Knockdown using shRNA was performed using the manufacturer's instructions (Santa Cruz). In gene knockdown using shRNA lentiviral particle transduction, cells were plated in 100 μL of 1640-RPMI (antibiotic-free) in 96-well plate at 5 × 10^4^/mL and incubated 12 h. Cells were exposed to media containing 1 μL of lentiviral particles and 5 μg/mL polybrene (Santa Cruz) for 24 h prior to maintenance in antibiotic-free 1640-RPMI. Cell viability and knockdown efficacy were assessed 72h after initial exposure to the lentivirus. To establish and maintain stable cell lines selecting for knockdown, cells were exposed to media containing 2 μg/mL puromycin (Santa Cruz). Media was replaced every three days or passaged if cells were >80% confluence. KLHL5 knockdown (Santa Cruz, Product Number: sc-89298-V) was compared to negative controls of scramble sequence (Product Number: sc-108080) as well as controls with no knockdown target.

The sequences used for gene knockdown using siRNA (Thermo Fisher) are as follows: (Sense: CAGG CCGCCUUGAAUUAAAtt) and (Antisense: UUUAAU UCAAGGCGGCCUGta). Negative control scramble siRNA designed to target no gene (Sense: CGUUAA UCGCGUAUAAUACGCGUat) and (Antisense: AUACGCGUAUUAUACGCGAUUAACGac).

siRNA was introduced into the cells using Lipofectamine^®^ 2000 (Thermo Fisher). Serum-free, antibiotic-free 1640-RPMI media was used during knockdown. 30 μL of Lipofectamine in 970 μL media and 60 μL of 20 μM siRNA in 940 μL media were incubated separately for five minutes, then combined and incubated for 20 minutes. This mixture was added to 8 mL of media containing 2.5 × 10^5^ cells/ml and plated in a 100 mm plate yielding a final siRNA concentration of 1.2 nM. After 16 hours incubation, cells were transferred to 96-well plates at 4000 cells per well in 100 μL of antibiotic-free, 10% FBS 1640-RPMI. Inner wells of the plate were used for experimentation with water-filled outer wells. For knockdown-drug combination studies, drugs were added six hours after plating into 96-well format.

Knockdown efficiency was tested using qRT-PCR (Applied Biosystems 7900-HT, Thermo Fisher). Gene expression levels were normalized to expression of the geometric mean of three reference genes ESD, MRPL19, and IPO8 [23]. Taqman probes (Life Technologies, Thermo Fisher) for each gene are as follows: KLHL5: Hs01567850_g1, ESD: Hs00382667_m1, MRPL: Hs00608519_m1, and IPO8: Hs00183533_m1.

### Data analysis

TCGA (The Cancer Genome Atlas) gene expression RNAseq data (IlluminaHiSeq: log2-normalized_count+1) was downloaded from Xena browser (https://xenabrowser.net/datapages/). The cancer types studied were: Urothelial Bladder Carcinoma (BLCA), Breast invasive carcinoma (BRCA), Colon adenocarcinoma (COAD), Esophageal carcinoma (ESCA), Head neck squamous cell carcinoma (HNSC), Chromophobe renal cell carcinoma (KICH), Renal clear cell carcinoma (KIRC), Renal papillary carcinoma (KIRP), Hepatocellular carcinoma (LIHC), Lung adenocarcinoma (LUAD), Lung squamous cell carcinoma (LUSC), Prostate adenocarcinoma (PRAD), Stomach adenocarcinoma (STAD), Thyroid carcinoma (THCA), and Uterine Corpus Endometrial Carcinoma (UCEC). The expression levels of forty KLHL genes were compared in these fifteen cancer types using statistical analyses.

NCI-60 data and Pearson correlations between microarray gene expression and drug effect on NCI-60 cell lines were obtained using data from CellMiner™ (https://discover.nci.nih.gov/cellminer/) [24].

Statistical analyses were performed using the R language and environment for statistical computing (R version 3.2.2; R Foundation for Statistical Computing; https://www.r-project.org/). Data was visualized using R or Tableau (Tableau 10.4; Tableau Software; https://www.tableau.com/). Prior to analysis, the normalized counts were log2 transformed to achieve a normal distribution. Fold change (FC) between cancer and adjacent normal samples as well as the *p*-values for these differences were calculated. The diagnostic power of individual genes to differentiate cancer patients and respective controls was assessed using the area under the curve (AUC) of the receiver operating characteristic (ROC) curves.

Cox proportional hazards models were used to evaluate the impact of gene expression levels on overall survival. Overall survival data (diagnosis to date of death) were obtained from the TCGA patient phenotype files. Patients who are alive with no evidence of disease were censored at the date of last follow-up visit. Kaplan–Meier survival analysis and log-rank test were used to compare differences in overall survival between groups. Classifications were made using different cut-offs of expression level at ten-percentile intervals, and the hazard ratio (HR) and *p*-value for each grouping were recorded.

Interacting proteins or enriched functions of interacting proteins were obtained from STRING database of known and suspected protein-protein interactions were imported via the STRING app (version 10.5; STRING Consortium; https://string-db.org/) in Cytoscape (Version 3.5.1; Cytoscape Consortium https://cytoscape.org/).

## SUPPLEMENTARY MATERIALS FIGURES AND TABLES


