# Supplementary material for: KLHL5 knockdown increases cellular sensitivity to anticancer drugs
Source: Oncotarget. 2018 Dec 21;9(100):37429–38. doi: 10.18632/oncotarget.26462 (PMC6324770; doi:10.18632/oncotarget.26462)
Supplement: Supplementary file 1 [file oncotarget-09-37429-s001.pdf]

KLHL5 knockdown increases cellular sensitivity to anticancer drugs

SUPPLEMENTARY MATERIALS

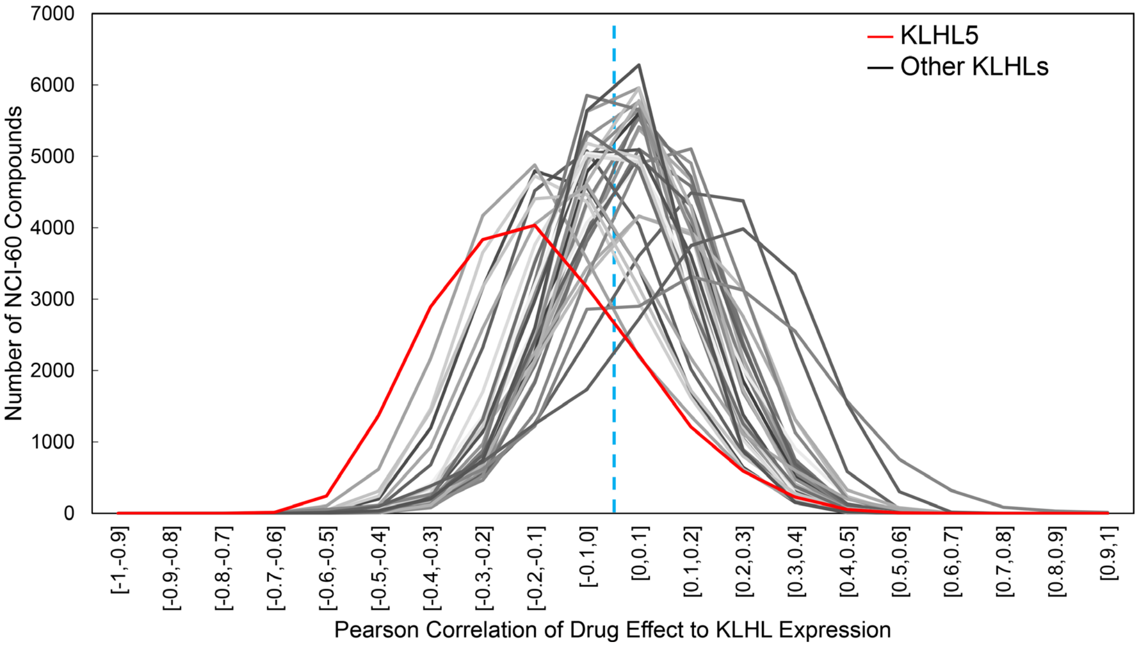

Supplementary Figure 1: Pearson correlations between KLHLs and anticancer compounds. Histogram for KLHLs indicates that KLHL5 possesses a high number of moderate or high negative correlations with compounds (< –0.4) and a distribution of correlations with the ~20,000 compounds that is shifted in distribution towards a negative correlation.

Mechanism	Drug Target	Drug Name	CL	[μM]
Apoptosis	Bcl-2	Navitoclax	O	1
			S	0.1
Cell Cycle	Aurora Kinase	AMG 900	O	0.1
			S	0.1
		Barasertib	O	0.1
		CYC116	O	1
		Danusertib	O	1
		ENMD-2076	O	1
		PF-03814735	S	0.1
		SNS-314 Mesylate	O	0.1
				1
		Tozasertib	O	0.1
	CDK	SNS-032	O	0.1
	Chk	AZD7762	S	0.01
Cytoskeletal Signaling	HSP	Ganetespib	S	0.01
		Geldanamycin	S	0.01
DNA Damage	DNA/RNA Synthesis	Fludarabine Phosphate	O	0.1
		Nelarabine	O	0.1
	Topoisomerase	Daunorubicin HCl	S	0.01
		Idarubicin HCl	O	0.1
		Teniposide	S	0.1
Endocrinology	ER/PR	Raloxifene HCl	O	0.1
Epigenetics	EGFR	CUDC-101	O	0.1
	HDAC	Entinostat	O	1
		PCI-24781	S	0.1
	Sirtuin	SRT1720	O	1
MAPK	MEK	TAK-733	S	0.1
		Trametinib	S	0.1
Metabolism	ALDH1	Disulfiram	O	1
NF-kB	E2 conjugating	BAY 11-7082	O	1
PI3K/Akt/mTOR	Akt	Triciribine	O	0.1
	PI3K	GDC-0941	O	0.1
			S	0.1
		XL147	O	0.1
		YM201636	O	0.1
		ZSTK474	S	0.1
	mTOR	AZD8055	S	0.01
		CH5132799	S	0.1
		Everolimus	O	1
		INK 128	S	0.01
		Ku-0063794	O	0.1
Proteases	Proteasome	Ixazomib	S	0.1
RTK and Angiogenesis	EGFR	Dacomitinib	O	1
			S	0.1
		Erlotinib HCl	O	0.1
		WZ4002	O	0.1
	FAK	PF-562271	O	1

DNA Damage	DHFR	Pemetrexed	O	0.1
Metabolism	Dehydrogenase	Mycophenolate mofetil	S	0.1
PI3K/Akt/mTOR	Akt	Triciribine	S	0.1
	mTOR	BEZ235	S	0.01
RTK and Angi..	Bcr-Abl	Rebastinib	S	0.1

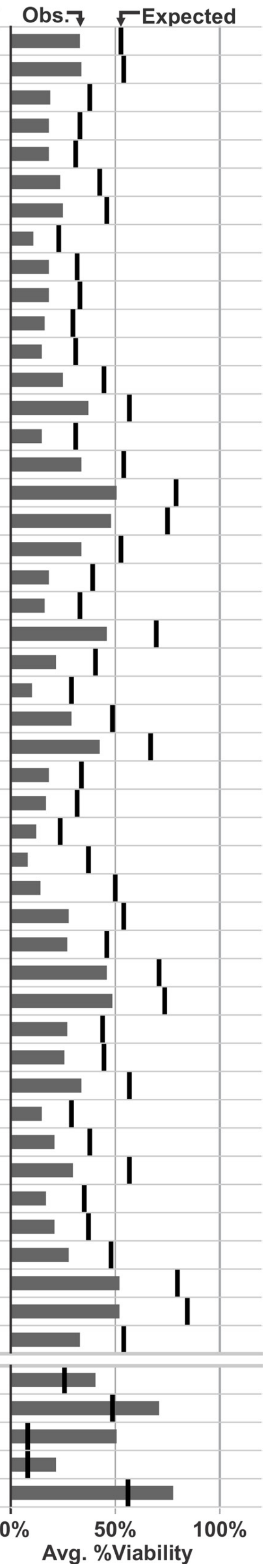

Supplementary Figure 2: Highly synergistic/antagonistic drugs with KLHL5 knockdown. Drugs with highest synergy or antagonism with KLHL5 knockdown. Cell line (CL) SN12C (S) was treated at 0.1 and 0.01 μM concentrations and OVCAR-8 (O) was treated at 1 and 0.1 μM concentrations. The viabilities upon treatment with compounds with the highest differences between the expected (knockdown only x drug only) and observed (knockdown and drug combined treatment) from the screening tests are shown.

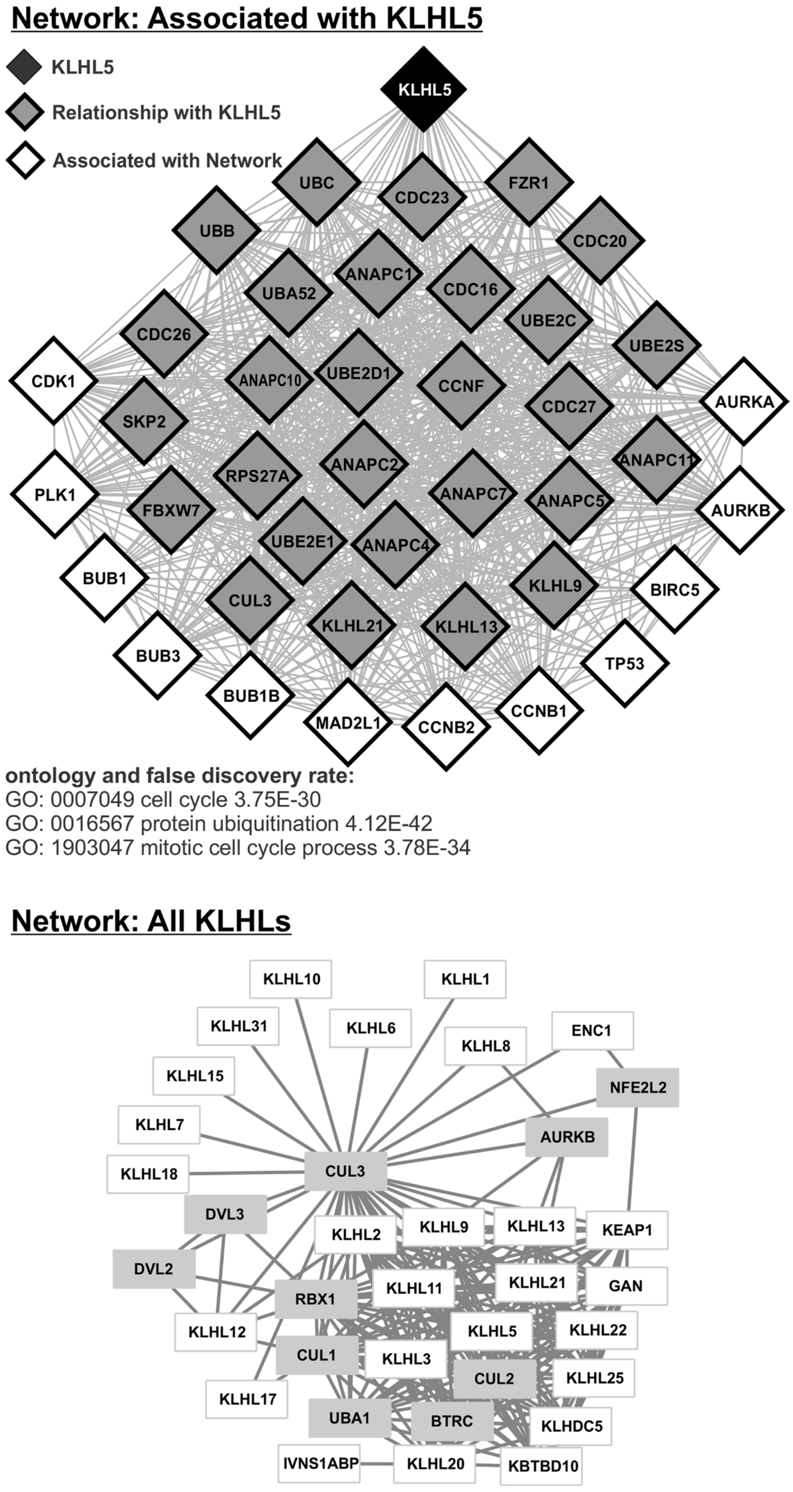

Supplementary Figure 3: STRING network analysis. String-db.org was consulted for information about interactions between KLHLs as well as proteins known or suspected to associate with KLHL5.
